# Lateralized behavior and cardiac activity of dogs in response to human emotional vocalizations

**DOI:** 10.1038/s41598-017-18417-4

**Published:** 2018-01-08

**Authors:** Marcello Siniscalchi, Serenella d’Ingeo, Serena Fornelli, Angelo Quaranta

**Affiliations:** Department of Veterinary Medicine, Section of Behavior Sciences and Animal Bioethics, University of Bari “Aldo Moro”, Bari, Italy

## Abstract

Over the recent years, the study of emotional functioning has become one of the central issues in dog cognition. Previous studies showed that dogs can recognize different emotions by looking at human faces and can correctly match the human emotional state with a vocalization having a negative emotional valence. However, to this day, little is known about how dogs perceive and process human non-verbal vocalizations having different emotional valence. The current research provides new insights into emotional functioning of the canine brain by studying dogs’ lateralized auditory functions (to provide a first insight into the valence dimension) matched with both behavior and physiological measures of arousal (to study the arousal dimension) in response to playbacks related to the Ekman’s six basic human emotions. Overall, our results indicate lateralized brain patterns for the processing of human emotional vocalizations, with the prevalent use of the right hemisphere in the analysis of vocalizations with a clear negative emotional valence (i.e. “fear” and “sadness”) and the prevalent use of the left hemisphere in the analysis of positive vocalization (“happiness”). Furthermore, both cardiac activity and behavior response support the hypothesis that dogs are sensitive to emotional cues of human vocalizations.

## Introduction

Evolutionary and ontogenetic processes played a pivotal role in dogs’ ability to detect social information from human behavior, providing the basis for complex forms of interspecific social communication^1,2^. There is a growing body of literature showing that dogs developed cognitive and social abilities in order to communicate with humans. Dogs are able to interpret human communicative gestures (e.g. the direction in which humans are facing or gazing), to detect his attentional states^3,4^ and to recognize different emotions by looking at human faces ^5,6^. Furthermore, recent studies reported that dogs discriminate human emotional faces from neutral ones^5,6^, that they are able to distinguish between happy and angry emotional human faces expressions^7^ and between the happy and neutral expression of the owner^8^. Moreover, Albuquerque *et al*.^9^ reported a cross-modal capacity in dogs related to the integration of visual and auditory emotional cues. In particular, dogs can correctly match “happy” or “angry” human faces with a vocalization expressing the same emotional valence.

Regarding the auditory sensory domain, it has been reported that dogs recognize the different valences of positive (laughing) and negative (crying) emotional sounds, showing an increase of indicators for arousal and negative emotional states in response to negative emotional sounds compared to positive ones^10^. Nevertheless, further studies are required in order to set reliable behavior indicators for positive emotional states in dogs.

To date, evidence for dogs’ perception and processing of human vocalizations characterised by different emotional valence is scarce^11^. Considering that it has been reported that the six basic emotions universally inferred from facial expressions^12^ are cross-culturally recognized from vocal signals in humans^13^, our study aimed at investigating if dogs are able to recognize the six basic emotions expressed by human non-verbal vocalizations.

In order to investigate this issue, we used the head-orienting paradigm to evaluate the potential asymmetrical behavior responses of dogs to human emotional vocalizations. The head-orienting response is commonly used as a behavior method for studying lateralized attention to acoustic stimuli in mammals tested in unrestrained conditions^14^. It requires sounds to be played simultaneously from two speakers located symmetrically at the same distance from the tested animal’s head. In order to ensure the correct position of the animal, the experiment is usually run during its feeding behaviors, positioning a bowl containing food midway between the two speakers. Since the head turning is an unconditioned response, its direction indicates the advantage of the contralateral hemisphere in processing the acoustic stimulus (e.g. if the subject turns his head towards the speaker with the right ear leading, the acoustic input is processed primarily by the left hemisphere, or at least in the initial attention to the stimulus^14–16^).

Specifically, in dogs a striking left head-orienting bias was observed in response to thunderstorm playbacks, confirming the right hemisphere advantage in attending to threatening and alarming stimuli^17^. On the contrary, conspecific vocalizations elicited a significant head-turning bias to the right (left hemisphere advantage). The specialization of the left hemisphere in processing the vocalizations of familiar conspecifics has been also reported for other animal models, such as nonhuman primates^18,19^, horses^20^, cats^21^, and sea lions^22^. Nevertheless, recent studies employing the orienting paradigm in other species found an inconsistent pattern of the head-turning response to conspecific calls. For instance, Vervet monkeys showed a right hemisphere dominant activity^23^ while no bias was found for Barbary macaques^24^. Moreover, a sex-specific asymmetries was shown for mouse lemurs, in particular male individuals displayed a left hemisphere bias in response to conspecific vocalizations with negative emotional valence^16^. This contradictory pattern might be due to a different phylogenetic distribution of hemispheric specialization and lateralization in closely related species^25^ or to the different emotional valence of the message conveyed. Furthermore, within the canine species, it has been reported that the left hemisphere involvement in attending to conspecific vocalizations depends on the characteristics of the sound, for example on the temporal acoustic features of the calls^26^. When dogs were presented with the reversed versions of specific vocalizations of play, disturbance and isolation, they showed a shift in their head-orienting behavior from a right-ear orienting bias (normal call versions) to a left-ear orienting bias (play calls) or to no asymmetry (disturbance and isolation calls^26^). In addition, recent studies describe a right hemisphere dominant activity to process conspecific vocalizations when they elicit intense emotions^17,27^.

Dogs show also an asymmetric head-turning behavior in response to human vocalizations. They displayed a significant bias to turn the head with the right ear leading (left hemisphere activity) when presented with a familiar spoken command in which the salience of meaningful phonemic (segmental) cues was artificially increased; on the other hand, they showed a significant head-turning behavior to the left side (right hemisphere dominant activity) in response to commands with artificially increased salience of intonational or speaker-related (suprasegmental) vocal cues^28^. Nevertheless, the more recent results of Andics *et al*.^29,30^ showed the opposite pattern of the hemispheres activity. Using the fMRI technique, they found a right hemisphere advantage in processing meaningful words and a left hemispheric bias for distinguishing intonationally marked words.

Overall, although these experiments showed lateralized auditory functions in the canine brain and provide insights into mechanisms of interspecific vocal perception, it remains unclear how dogs perceive and process the six basic emotions expressed by human non-verbal vocalizations. One of the possible methods employed to assess brain emotional functioning in the animal kingdom consists in observing and analyzing physiological (e.g. cardiac activity) and behavior responses to specific stimuli in experimental conditions that resemble as much as possible the natural ones^31^. Regarding the physiological response, there is now scientific evidence that cardiac activity could be considered a valid indicator to predict different emotional states in dogs^32–35^.

As to the behavior response, a recent study scored dogs’ behaviors in order to investigate emotional contagion to conspecific and human emotional sounds^10^. Although results indicate that for both canine and human sounds dogs express more stress behaviors after hearing sounds with a negative emotional valence, further studies are required to determine valid and reliable behavior indicators for positively valenced sounds^10^.

The study of behavior lateralization has the potential to provide new insights into animal emotional processing^36^. An increasing body of evidence shows common lateralized neural patterns for emotional processing across all vertebrate classes, with specialization of the right hemisphere for processing withdrawal and negative emotions (e.g. fear and aggression) and a dominant role of the left hemisphere for processing positive emotions and approach^37,38^. Thus, external manifestation of hemispheric dominance (e.g. head-turning behavior) matched with both behavior and physiological responses could represent useful tools for understanding the valence of an emotion perceived by an animal during a particular situation, facilitating the categorization of the emotion along with valence and arousal dimensions^39–41^. In the light of this evidence, the aim of the present work was to investigate dogs’ emotional responses to human non-verbal emotional vocalizations by measuring subjects’ head-turning bias (valence dimension) and the related behavior and cardiac activities (arousal dimension).

## Results

### Head-orienting response

Friedman’s ANOVA revealed that there was no effect of acoustic stimulus on the % of response (χ2 (5) = 6,782, P = 0.237); average %: anger (83.3%); fear (80.0%); disgust (93.3%); sadness (76.6%); surprise (90.0%) and happiness (93.3%).

**Figure 1:**
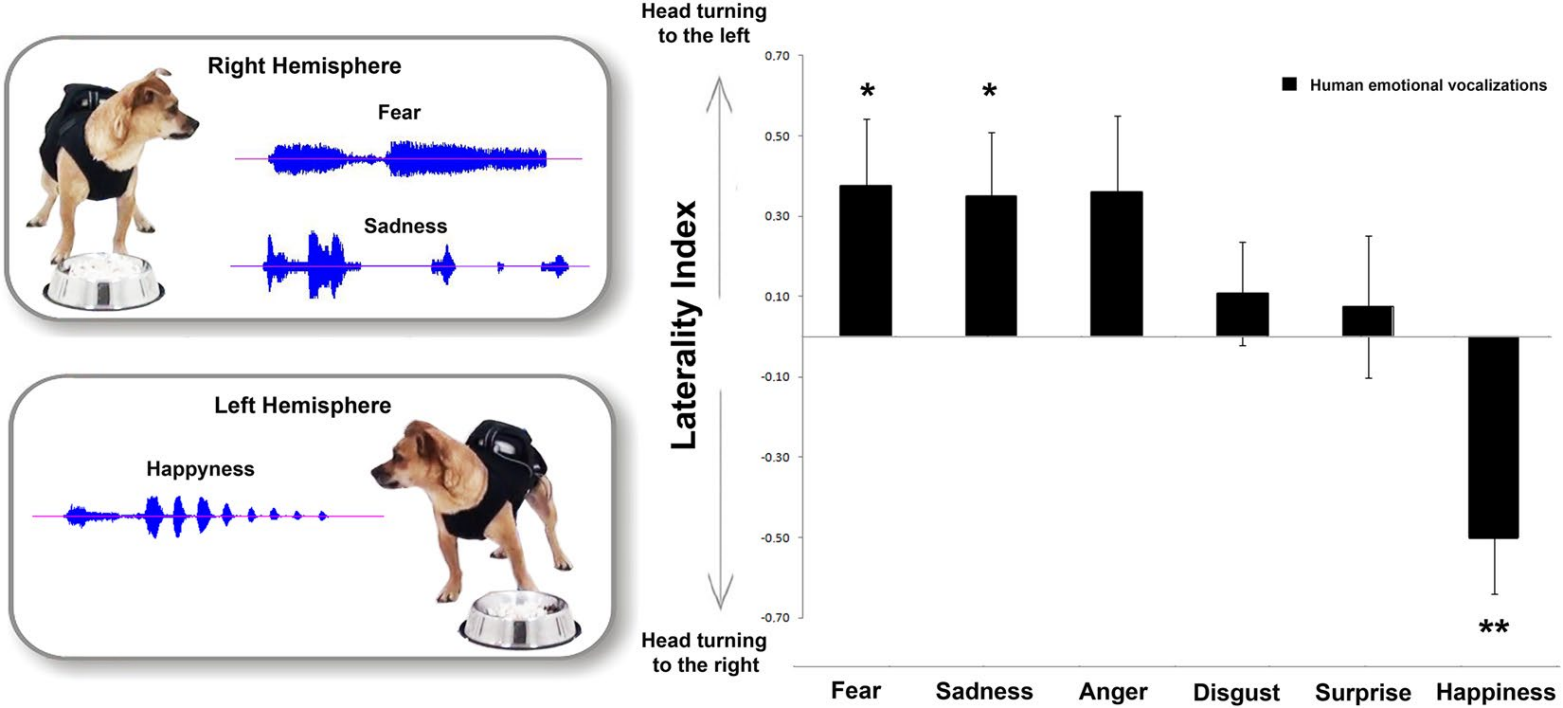
Head orienting response to different vocalizations. Laterality index for the head-orienting response of each dog to playbacks: a score of 1.0 represents exclusive head turning to the left side and −1.0 exclusive head turning to the right side (group means with SEM are shown); Asterisks indicate significant biases. *P < 0.05; **P < 0.01 (One-Sample Wilcoxon Signed Ranks Test).

Results for the head-orienting response are shown in Fig. 1. A significant main effect of playbacks was observed (F(5,99) = 5.766, P = 0.000; GLMM analysis): pairwise comparisons revealed that this main effect was due to “happiness” vocalization being significantly different from other sounds (“happiness” vs. “fear”, “anger” and “sadness” (P = 0.000); “happiness” vs. “disgust” (P = 0.009) and “surprise” (P = 0.026); Fisher’s LSD). In addition, the analysis revealed that for “fear” and “sadness” call types, dogs consistently turned their head with the left ear leading (“fear”: Z = 140.000, P = 0.039; “sadness”: Z = 102.000, P = 0.046; One-Sample Wilcoxon Signed Ranks Test) (see Fig. 1). A very similar trend was observed for “anger” vocalization even if not statistically significant (Z = 221.000, P = 0.072). On the other hand, a significant right bias in the head turning response was found when dogs attended to playbacks of “happiness” (Z = 46.000, P = 0.003). No statistical significant biases were found for both “disgust” and “surprise” stimuli (P > 0.05, One-Sample Wilcoxon Signed Ranks Test). On investigating possible methodological confounding factors, a binomial GLMM analysis revealed that the direction of head orienting response turns was not significantly influenced by vocalization gender (F(1,99) = 0.102, P = 0.750) and playback order (F(6,93) = 0.705, P = 0.646). In addition no effects of sex (F(1,99) = 0.306, P = 0.581), age (F(1,99) = 0.000, P = 0.998) and temperament questionnaire scores were observed (P > 0.05 for all queries of the questionnaire, see Table 1).

**Table 1:** Questionnaire queries. Effects of questionnaire queries on “Orienting Response”, “HV”, “LV”, “AUC”, “AAC” and “Stress” variables (df1 = 1; df2 = 136; GLMM analysis).

Queries	Orienting Response		HV		LV		AUC		AAC		Stress	
	F	P	F	P	F	P	F	P	F	P	F	P
(1) Stranger-directed aggression	0.272	0.603	0.362	0.548	0.593	0.443	3.455	0.065	0.775	0.380	5.185	0.001
(2) Owner-directed aggression	1.427	0.235	3.905	0.050	11.865	0.001	0.006	0.938	0.163	0.687	0.000	0.982
(3) Stranger-directed fear	1.061	0.360	1.876	0.173	0.942	0.334	0.275	0.601	3.083	0.081	0.054	0.816
(4) Non social fear	0.060	0.807	0.138	0.711	4.232	0.052	5.862	0.017	2.652	0.106	4.331	0.045
(5) Separation-related behaviour	0.203	0.654	0.874	0.352	6.394	0.013	4.085	0.055	0.044	0.834	0.412	0.522
(6) Attachment or attention-seeking behaviour	0.418	0.519	0.032	0.858	0.302	0.584	5.521	0.045	0.216	0.643	1.157	0.284
(7) Trainability	0.701	0.404	0.582	0.447	0.900	0.344	4.394	0.038	6.686	0.011	0.000	0.996
(8) Excitability	0.629	0.430	0.755	0.386	0.826	0.365	0.083	0.774	0.449	0.504	3.189	0.076
(9) Pain sensitivity	0.362	0.549	3.730	0.056	3.050	0.083	0.025	0.874	0.005	0.945	0.006	0.936

### Reactivity and latency to resume feeding

**Figure 2:**
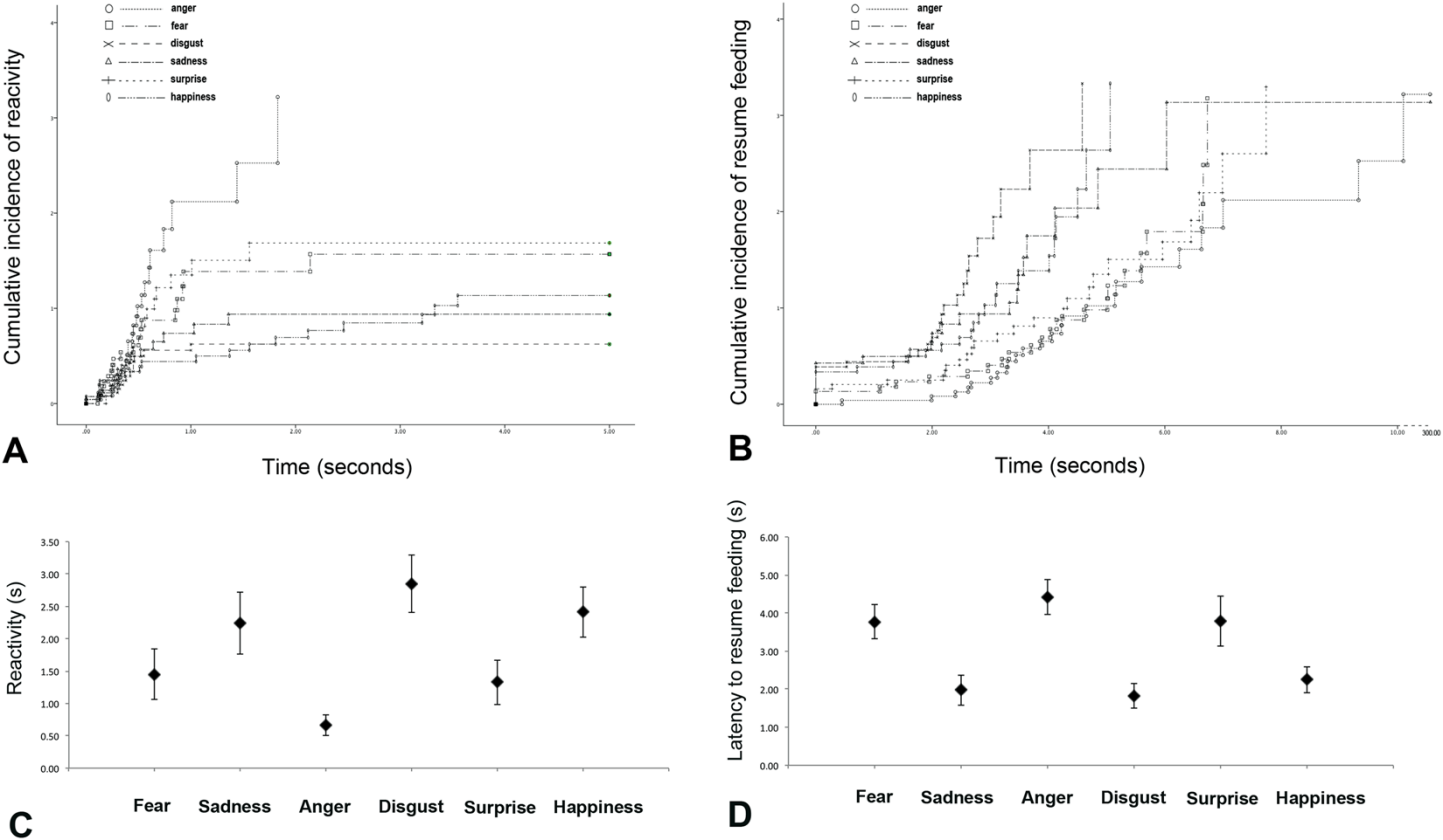
Reactivity and latency to resume feeding. The cumulative incidences of reactivity (**A**) and latency to resume feeding (**B**) during playbacks’ presentations.

The cumulative incidences of reactivity and latency to resume feeding during playbacks’ presentations are presented in Fig. 2(A,B). As for reactivity, mixed effects Cox regression revealed that subjects hearing “anger” playbacks had a higher probability to react than after attending, respectively, playbacks of happiness (β(SE) = 0.93(0.37); [Exp(β) = 0.39; 95%-CI = 0.19;0.81]; P = 0.012), “disgust” (β(SE) = 1.16(0.43); [Exp(β) = 0.31; 95%-CI = 0.13;0.74]; P = 0.008) and “sadness” (β(SE) = 1.13(0.39); [Exp(β) = 0.32; 95%-CI = 0.15;0.69]; P = 0.004) see Fig. 2(A–C). The probability to react to the stimuli was increased in male subjects with respect to females (β(SE) = 0.52(0.24); [Exp(β) = 0.59; 95%-CI = 0.37;0.95]; P = 0.031) while it was decreased as age increased (B(SE) = −0.1(0.047); [Exp(β) = 0.90; 95%-CI = 0.82;0.99]; P = 0.033). A primary effect of the first playback has been observed since the probability to react was increased if the sound was presented after respectively “anger” (β(SE) = 0.69(0.34); [Exp(β) = 1.99; 95%-CI = 1.03;3.85]; P = 0.041) and “surprise” acoustic stimuli (β(SE) = 0.77(0.37); [Exp(β) = 2.17; 95%-CI = 1.04;4.5]; P = 0.038); on the other hand, the probability to react was lower if the sound was presented after “sadness” stimulus (β(SE) = −1.51(0.54); [Exp(β) = 0.22; 95%-CI = 0.07;0.63]; P = 0.005). Finally, there were effects of stress behaviors and query scales, since the probability to react to playbacks increased with increasing scores of stress behaviors during the test (β(SE) = 0.25(0.08); [Exp(β) = 1.28; 95%-CI = 1.08;1.51]; P = 0.003) and increasing scores of excitability in the query (β(SE) = 0.07(0.03); [Exp(β) = 0.39; 95%-CI = 1.00;1.14]; P = 0.048). Finally, the probability to react to vocalizations sounds decreased with increasing scores of “stranger aggression” in the query (β(SE) = −0.06(0.02); [Exp(β) = 0.94; 95%-CI = 0.89;0.99]; P = 0.019).

As for the latency to resume feeding, mixed effects Cox regression revealed that dogs hearing “anger” had smaller probability to resume feeding than when they were hearing respectively “happiness” (β(SE) = −1.30(0.33); [Exp(β) = 3.70; 95%-CI = 1.91;7.15]; P = 0.000), “fear” (β(SE) = −0.73(0.32); [Exp(β) = 2.07; 95%-CI = 1.10;3.90]; P = 0.023), “disgust” (β(SE) = −1.28(0.34); [Exp(β) = 3.59; 95%-CI = 1.84;7.05]; P = 0.000) and “sadness” (β(SE) = −1.15(0.35); [Exp(β) = 3.16; 95%-CI = 1.58;6.33]; P = 0.001) (see Fig. 2B–D). The probability to resume feeding after hearing the sounds decreased as age increased (β(SE) = −0.08(0.04); [Exp(β) = 0.91; 95%-CI = 0.85;0.98]; P = 0.024). As expected, there were effects of stress behaviors and query scales, since the probability to resume feeding decreased with increasing scores of stress behaviors during the test (β(SE) = −0.28(0.08); [Exp(β) = 0.75; 95%-CI = 0.64;0.88]; P = 0.001) and increasing scores of respectively trainability (β(SE) = −0.06(0.03); [Exp(β) = 0.94; 95%-CI = 0.88;0.99]; P = 0.042), excitability (β(SE) = −0.05(0.02); [Exp(β) = 0.94; 95%-CI = 0.90;0.99]; P = 0.040) and pain sensitivity (β(SE) = −0.11(0.04); [Exp(β) = 0.89; 95%-CI = 0.81;0.98]; P = 0.019) in the query.

### Cardiac activity

**Figure 3:**
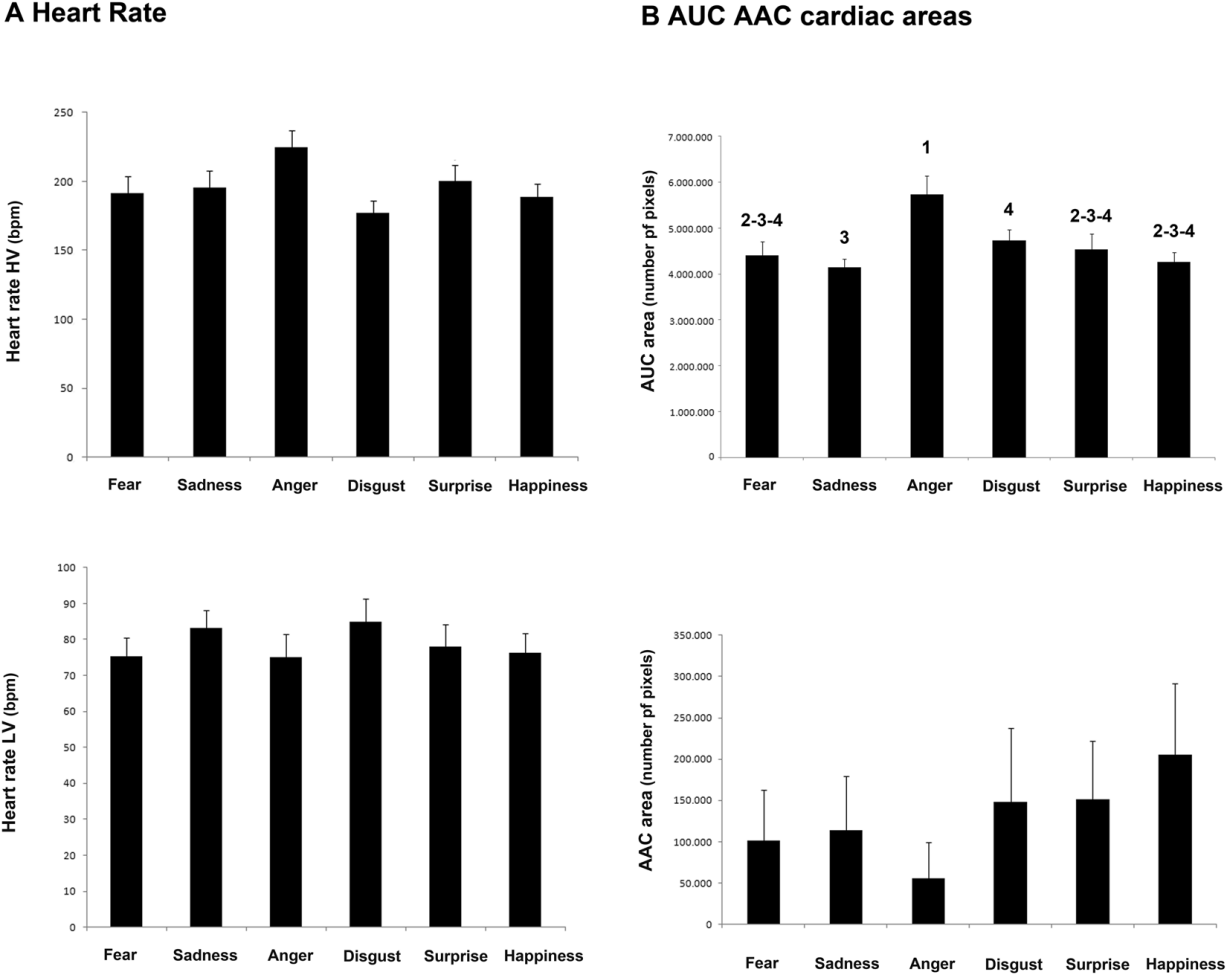
Cardiac activity. (**A**) Highest value (HV) and lowest value (LV) of the dogs’ heart rate (HR) in response to presentation of different human vocalizations (means with S.E.M. are shown). (**B**) The Areas Under Curve (AUC; **A**) and Above Curve (AAC; **B**) in response to presentation of human vocalizations (means with S.E.M. are shown); Different numbers indicate statistical significance according to Fisher’s LSD test.

Results for cardiac activity are shown in Fig. 3. The highest (HV) and lowest values (LV) of the Heart Rate (HR) response to different playbacks were analyzed. Moreover, the area delimited by the HR curve and the HR basal average (baseline) was computed for each dog and the Area Under Curve (above baseline and under curve, AUC) and the Area Above Curve (under baseline and above curve, AAC) were then obtained. No statistically significant differences were observed between acoustic stimuli regarding higher heart rate values (GLMM analysis): emotional category: (F(5,131) = 1.449, P = 0.211; see Fig. 3A); playback order (F(6,131) = 0.966, P = 0.451); vocalization gender (F(1,131) = 0.419, P = 0.518); sex (F(1,131) = 0.023, P = 0.881); age (F(1,131) = 3.431, P = 0.066;) and questionnaire scales (P > 0.05 for all queries of the questionnaire, see Table 1).

Regarding lower heart rate values (see Fig. 3A), a statistically significant effect of age was observed F(1,131) = 6.701, P = 0.011; GLMM analysis), showing that adult subjects have higher rate to have lower LV values after attending to emotional playbacks (β(SE) = −2.44(0.94); [95%-CI = −4.30; −0.57]). In addition, the analysis revealed that subjects with higher scores for both “owner aggression” (β(SE) = 6.97(2.02); [95%-CI = 2.97;10.98]; P = 0.001) and “separation related behaviors” (β(SE) = 1.46(0.58); [95%-CI = 0.31;2.61]; P = 0.013) queries had lower values of LV after hearing emotional vocalizations (see Table 1). No other statistically significant effects were observed regarding lower heart values: emotion category (F(5,131) = 0.796 P = 0.554; GLMM analysis); vocalization gender (F(1,131) = 0.136, P = 0.712); playback order (F(6,131) = 0.960, P = 0.493); sex (F(1,131) = 1.379, P = 0.242); questionnaire scales (P > 0.05 for all others queries of the questionnaire, see Table 1).

A significant main effect of playbacks was observed in the overall increase of the heart rate values compared to the baseline (see Fig. 3B, AUC) (i.e. the area above baseline and under curve (F(5,131) = 4.242, P = 0.001) after controlling for the effect of playback order (F(6,131) = 1.485, P = 0.188) and vocalization gender (F(1,131) = 1.586, P = 0.210) (GLMM analysis): pairwise comparisons revealed that the AUC values were higher for “anger” stimulus than for the other emotional vocalizations: “anger” vs. “sadness” (P = 0.000); “anger” vs. “happiness” (P = 0.001); “anger” vs. “fear” (P = 0.002); “anger” vs. “surprise” (P = 0.017) and “anger” vs. “disgust” (P = 0.049). In addition, the analysis revealed that “disgust” stimulus induced higher AUC values than “sadness” (P = 0.008). No effects of sex (F(1,131) = 0.096, P = 0.757) and age (F(1,131) = 1.761, P = 0.187) were found. As to the questionnaire, the analysis revealed a statistically significant effect of query 6 indicating that the higher the scores for “attachment or attention-seeking behaviors”, the more likely dogs had lower AUC values after attending vocalizations (β(SE) = −52.877(26.104); [95%-CI = −104.518;−1.235]; P = 0.045); on the other hand, subjects with higher scores for “non-social fear” (β(SE) = 75.632(31.393; [95%-CI = 13.529;137.736]; P = 0.017) and “trainability” (β (SE) = 72.847(34.963); [95%-CI = 3.681;142.013]; P = 0.038) had higher AUC values after hearing emotional playbacks. No other statistically significant effects were found (P > 0.05 for all the remaining queries of the questionnaire, see Table 1).

Regarding the overall decrease of the heart rate values compared to the baseline (i.e. the area under baseline and above curve, AAC), the GLMM analysis revealed that the higher the scores for trainability, the more likely dogs had lower AAC values (β(SE) = −27.611(10.678); [95%-CI = −48.736; −6.487]; P = 0.011) (see Table 1).

No other statistical significant effects were observed in AAC values: emotional category (F(5,131) = 0.304, P = 0.910; GLM analysis) (Fig. 3B); vocalization gender (F(1,131) = 0.006, P = 0.941); playback order (F(6,131) = 0.928, P = 0.477); sex (F(1,131) = 0.806, P = 0.371); age (1,131) = 0.237, P = 0.627) and query scales (P > 0.05 for all the rest queries of the questionnaire, see Table 1).

### Behavior score

**Figure 4:**
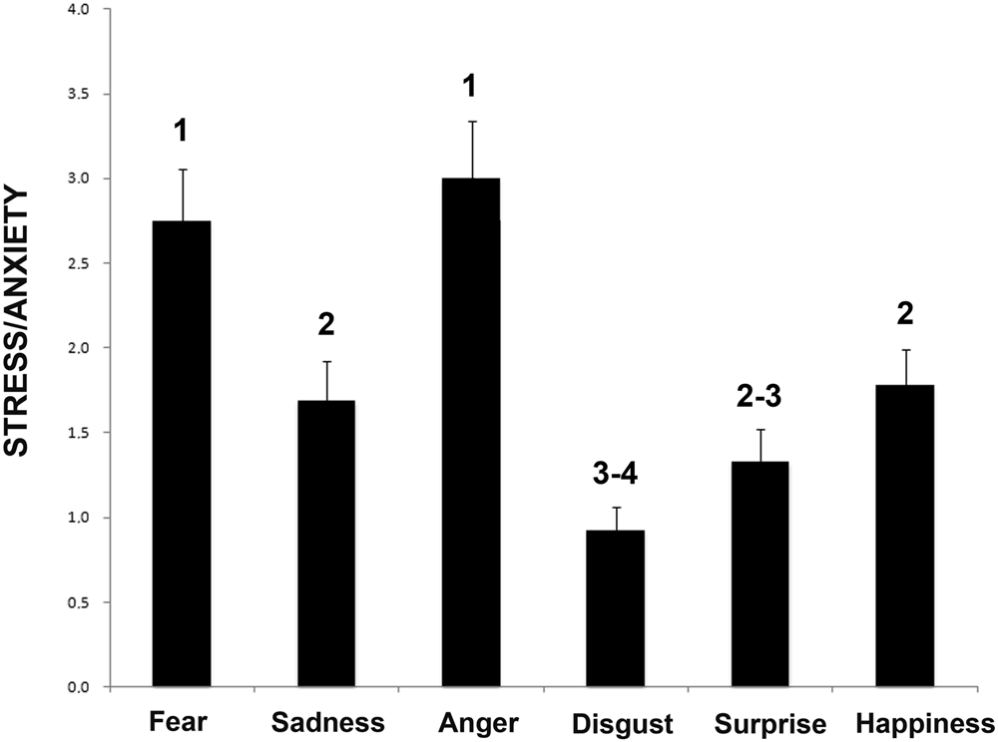
Behavioral score. Data for the score of the stress/anxiety behavioral category from the behavioral score for each dog during presentation of different playbacks (means with S.E.M. are shown); Different numbers indicate statistical significance according to Fisher’s LSD test.

As to behavioral score, analysis of the stressed behavioral category revealed that there was a significant difference between acoustic stimuli (F(5,131) = 10.851, P = 0.000; GLMM analysis, see Fig. 4) after controlling for the effect of both playback order (F(6,131) = 0.840, P = 0.541), vocalization gender (F(1,131) = 2.128, P = 0.147) and age (F(1,131) = 0.420, P = 0.518). A statistically significant effect of sex was observed (F(1,131) = 4.994, P = 0.027) indicating that male subjects have lower rate compared to females to display lower rate to display stress behaviors after attending to emotional playbacks (β(SE) = −0.44(0.19); [95%-CI = −0.82; −0.05]; P = 0.027). Post hoc pairwise comparisons revealed that dogs showed more stress-related behavior when they attended to “anger” and “fear” playbacks than to the others sounds, (“anger” “vs. “surprise” and “disgust” (P = 0.000); “anger” vs. “sadness” (P = 0.001); “anger” vs. “happiness” (P = 0.004); “fear” vs. “disgust” and “surprise” (P = 0.000); “fear” vs. “sadness” (P = 0.002); “fear” vs. “happiness” (P = 0.004); Fisher’s LSD). In addition stressed behavioral score was higher for “happiness” than “disgust” (P = 0.006). Finally, pairwise comparisons revealed higher stress behavioral scores while hearing the playbacks of “sadness” than “disgust” (P = 0.026) (see Fig. 4). Significant positive relationships were found between stress levels and queries 1 (β(SE) = 0.05(0.02); [95%-CI = 0.01;0.09]; P = 0.007) and 4 (β(SE) = 0.05(0.04); [95%-CI = 0.00;0.10]; P = 0.045; GLMM analysis) of temperament questionnaire scores indicating that the stronger the “aggressiveness to strangers” and “non social fear”, the more likely dogs have higher stress levels when attending to playbacks sounds (see Table 1).

Finally, tail-wagging behavior was observed during five occasions and 3 of these occurred after “surprise” and 2 after “happiness” sounds. In addition, after “surprise” playbacks dogs approached the speakers 2 times (given the low frequency of these observed behaviors, statistical analysis was not performed).

## Discussion

Previous studies have reported that dogs’ olfactory system works in an asymmetrical way to decode different emotions conveyed by human odors^32^. Our results demonstrate that this asymmetry is also manifested in the auditory sensory domain since dogs showed an asymmetrical head-orienting response to the playbacks of different human non-verbal emotional vocalizations. In particular, they turned the head with their left ear leading in response to “fear” and “sadness” human vocalizations. Given that in the head-orienting paradigm the head-turning direction indicates an advantage of the contralateral hemisphere in processing sounds^14^, the left head turning in response to “fear” and “sadness” vocalizations here reported suggests the prevalent activation of the right hemisphere. This finding is consistent with the general hypothesis of the right hemisphere dominant role in the analysis of intense emotional stimuli (e.g. horse^42–44^; dog^45^). Other evidences come from studies on cats, showing that, using the same head-orienting paradigm, they turned the head with their left ear leading in response to dogs’ “disturbance” and “isolation” vocalizations^21^.

**Figure 5:**
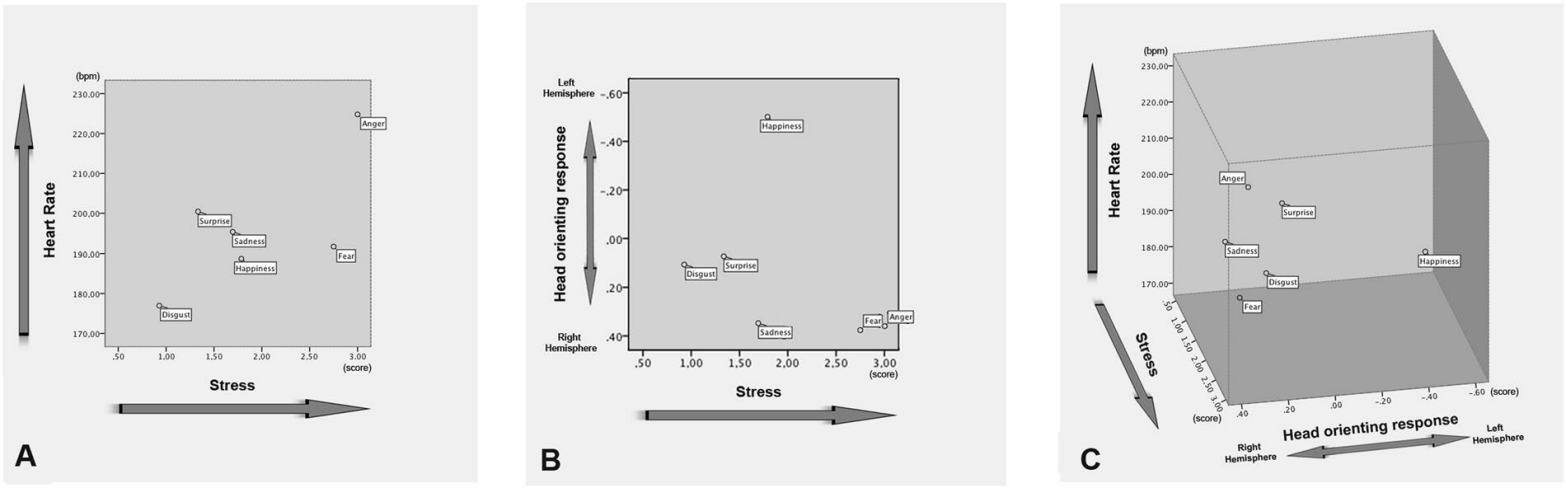
Scattergrams of (**A**) Arousal and (**B**,**C**) Valence dimensions. Data for the score of the arousal (higher heart rate and stress/anxiety behavioral category) and valence (head orienting response) dimensions of different playbacks (means are shown).

Furthermore, dogs’ right hemisphere activation to process stimuli of negative emotional valence has also been reported by studies on motor functions (e.g. tail wagging behavior, see Siniscalchi *et al*.^35^) and on sensory domains (e.g. vision^46^; olfaction^47^). Specifically, a bias to the left side (right hemisphere) in the head-turning response has been observed when dogs were presented with visual alarming stimuli (i.e. black silhouette of a snake and of a cat displaying an agonistic aversive posture^46^) and a right nostril preferential use (right hemisphere) to investigate conspecific “isolation” odours^32^. Our data from the arousal dimension indicate that although both “sadness” and “fear” vocalizations are processed mainly by the right hemisphere, after hearing “sadness” playbacks dogs were less stressed than after hearing “fear” (see scattergrams, Fig. 5). The latter could be explained by the fact that despite both “fear” and “sadness” vocalizations are characterized by negative valence, they can differ on the functional and communicative level. In some individuals, “sadness” vocalizations could be clearly an approach evoking call while “fear” vocalizations could produce a different reaction in the receiver (approach/withdrawal) depending on the social context in which it is produced and perceived. However, considering the communicative function of these vocalizations, it could be hypothesized that the “fear” ones may elicit stronger reactions in the listener, explaining the higher arousal and stress behaviors registered in response to this vocalization. Moreover, in the light of recent findings^48,49^, the higher arousal and stressed behaviors showed by dogs after hearing “fear” vocalizations, which is a higher-arousal emotion compared to “sadness”, suggest the occurrence of a cross-species emotional contagion between human and dogs. Nevertheless, further investigations are needed to address this issue.

As Fig. 3 shows, there was a clear tendency for dogs to turn their head to the left side in response to “anger” playbacks, but it didn’t reach statistical significance. Previous studies hypothesized that dogs perceive the “anger” emotion to have a negative emotional valence^50^. It has been recently reported indeed that dogs showed a left gaze bias while looking at human negative facial expressions (angry faces), suggesting the right hemisphere involvement in processing the emotional message conveyed^51^. Furthermore, dogs looked preferentially at the lower face region of unfamiliar humans showing a negative expression (“sadness” and “angry”), avoiding consequently an eye contact with a potential threatening stimulus^50^. The high emotional valence attributed to the anger emotion is also attested by the longer time employed to correctly associate a reward to a human angry face rather than a happy one^7^. One possible explanation for the weaker left orienting bias observed in response to the “anger” vocalizations with respect to “fear” and “sadness”, is that these sounds displayed an acoustic feature resembling the one of canine “threatening growls” (harsh, low frequency call). Although the emotional valence of this canine vocalization is similar to the “anger” one (most likely eliciting a right hemisphere activity), overall, a specialization of the left hemisphere for processing conspecific vocalizations has been observed^17^. In addition, fMRI studies identified two auditory regions in the dog brain, one bilaterally located and the other one in the left dorsal auditory cortex, both responding selectively to conspecific sounds^52^. Hence, it cannot be entirely ruled out the possibility that some subjects might have misinterpreted the “anger” vocalizations, categorizing them as a conspecific call. As a consequence, this phenomenon might have produced a sort of left hemisphere “interference” in processing the sound. On the other hand, as results for the head orienting response to “anger” sounds were marginally significant, it would be interesting to test this condition in future studies using a larger sample of dogs in order to verify if the lack of statistical significance is only a question of statistical power.

Regarding the “happiness” vocalization”, a clear right bias in the head-orienting response (left hemisphere advantage) was observed. Previous studies have reported a left-hemisphere specialization for approach behavior^53^. Specifically in dogs, a left-brain activation was indirectly observed throughout asymmetric tail wagging movements to the right side in response to stimuli that could be expected to elicit approach tendencies, such as seeing the owner^11^. Thus, the involvement of the left hemisphere in the analysis of “happiness” vocalizations suggests that dogs perceived this sound as an expression of a positive emotional state that could elicit approaching behaviors, having a central role in the beginning and maintaining the dog-human interaction (note that tail wagging behaviors were observed during “happiness” playbacks). This evidence is supported by recent fMRI studies indicating a left bias for more positive human sounds^52^ and an increase of functional connectivity in the left hemisphere in response to positive rewarding speech compared to neutral one^29^.

Overall, results from latency to resume feeding, cardiac activity and stress levels suggested that hearing “happiness” vocalization induced, as expected, low arousal levels with respect to hearing “fear” and “anger” but not “sadness”. The latter suggests that relying solely on the arousal dimension would not make it clear to distinguish between the emotions conveyed by sadness and happiness vocalizations (see scattergrams, Fig. 5). In dogs, this hypothesis is supported by recent findings that indicate that parasympathetic deactivation (i.e. arousal increasing) is associated with a more positive emotional state elicited by different positive stimuli (food or social rewards^33^).

Regarding “surprise” and “disgust” vocalizations, we found no biases in dogs’ head-turning response. This result may suggest that the dogs perceived these sounds to be less distinguishable than the others in terms of both emotional valence and degree of familiarity. In particular, concerning the “disgust” vocalizations, our results fit in with the hypothesis of Turcsàn *et al*.^8^ about the ambiguous valence that this emotion could have for dogs. In everyday life, different objects or situations eliciting a “disgust” emotion in the owner could be attractive for the dog (e.g. feaces) or, on the contrary, could be associated with a negative outcome (e.g. scolding). Thus, dogs’ behavior responses (approaching or withdrawal) and the emotional valence attributed (negative or positive) could be strictly dependent on the individual experiences. Regarding surprise, evidence from human studies reported that this emotion could be perceived as both positive and negative, depending on the goal conduciveness of the surprising event^54^ (note that in our experiments, during hearing surprise sounds, although the arousal levels were similar to those observed in response to sadness, tail wagging behavior and approaching behaviors to the speaker were observed). More interestingly, recent cognitive and psychophysiological studies indicate the possibility that surprise may be a (mildly) negative emotion^55^. The latter would be very similar to the slight left orienting (but not statistically significant) bias (right-hemisphere activation) observed here in dogs.

Overall, our results provide evidences about the existence of an emotional modulation of the dog brain to process basic human non-verbal emotional vocalizations. In particular, results from our experiments have shown that dogs process human emotional vocalizations in an asymmetrical way, predominantly using the right hemisphere in response to vocalizations with a clear negative emotional valence (i.e. “fear” and “sadness”) and the left hemisphere in response to “happiness” playbacks. In addition, both cardiac activity and behavior response support the hypothesis that dogs are sensitive to emotional cues of human vocalizations, indicating that coupling the use of valence and arousal dimensions is a useful tool to deeply investigate brain emotional functioning in the animal kingdom.

## Materials and Methods

### Subjects

Thirty-six domestic dogs of various breeds were recruited for this study. We excluded 6 dogs: two dogs, because they showed distress soon after entry into the room; two dogs did not respond to any playbacks (i.e. did not stop feeding behavior); one dog was influenced by the owner during the test; one dog due to procedural problem (connection lost between the cardiac wireless system for telemetric measurements and the computer). Hence the final sample consisted of 14 males (3 neutered) and 16 females (6 neutered) whose ages ranged from 1 to 13 years (3.90 ± 2.83; mean ± S.D.; see Suppl. Table 1). All subjects were pets living in households. To join the study, dogs were required to be food motivated, healthy and experimentally naïve. They also had to fast for at least 8 hours before the testing session. Before the experiment begun, clinical and audiological evaluation for hearing impairment were performed on all the sample by two veterinarians of the Department of Veterinary Medicine, University of Bari. None of the tested dogs had hearing impairment.

### Stimuli

**Figure 6:**
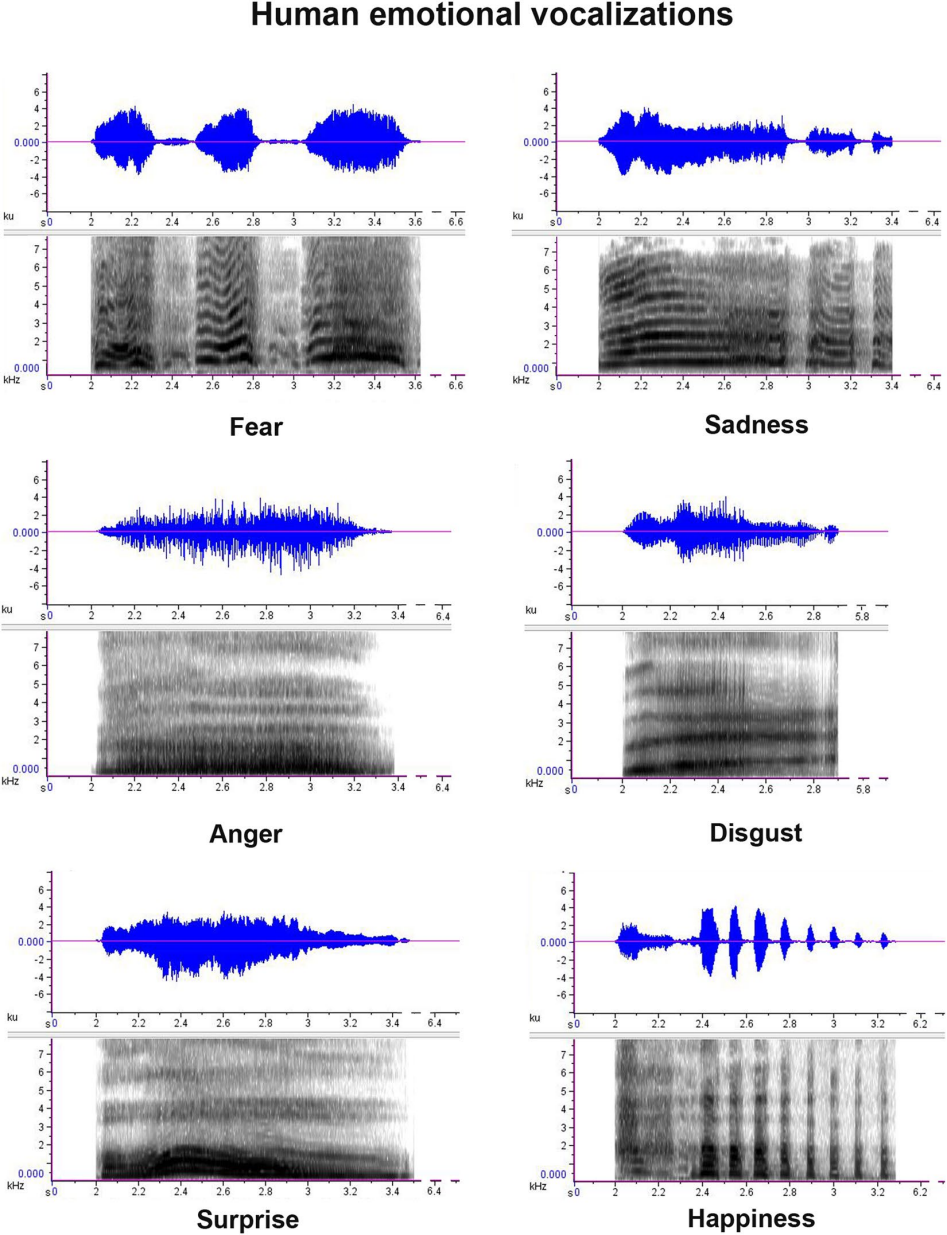
Spectrograms. Spectrograms’ samples of human vocalizations with different emotional valence used as playbacks.

Seven men and seven women, aged between 24 and 37 years, were asked to pronounce a set of non-verbal vocalizations, each expressing one of the six basic emotions^33^: happiness, surprise, disgust, fear, sadness and anger. According to Sauter *et al*.^13^, happiness sounds were laughs, disgust sounds were retches, fear sounds were screams, sadness sounds were sobs and anger sounds were growls. Surprise sounds were strong expirations producing “oh” vocalizations (see Fig. 6).

The sounds were produced in an anechoic chamber and each vocalization was digitally recorded employing Roland Edirol R-09HR, at a 24-bit quantization and 96 kHz sampling rate. The recordings were done in mono in order to avoid possible left-right asymmetries during playbacks.

Each acoustic stimulus was edited using Audition 2.0 (Adobe Inc.) so that it contained about 1 second of sound (vocalization) preceded and followed respectively by 2 s and 3 s of silence. Furthermore stimuli were equalized and their amplitude were homogenized in order to reach an average loudness of 69 dB when measured from the dog’s position. In addition recordings were filtered to remove background noises. Protmex MS6708 Portable Digital Decibel Sound Level Meter was used to ensure that the speakers broadcast at the same volume.

In order to select the most significant and clear vocalizations, all recordings were then presented to 10 volunteers, five men and five women, aged between 20 and 30 years, in a casual order but identical between subjects, and played at constant volume. After listening to each auditory stimulus, they were asked to fill in a questionnaire, indicating if it expressed a positive or negative emotion, which of the six basic emotions it represented and rating on a 3-point-scale how clearly they perceived the emotion conveyed (see Table 2 supplementary materials). A sub-sample of 18 vocalizations (three x each basic emotion) was then selected according to questionnaire results, so that three sets of the six emotional vocalizations were obtained (see supplementary material for the criteria selection, Suppl. Table 2 and emotional vocalizations sets’ details, Suppl. Table 3).

### Apparatus

**Figure 7:**
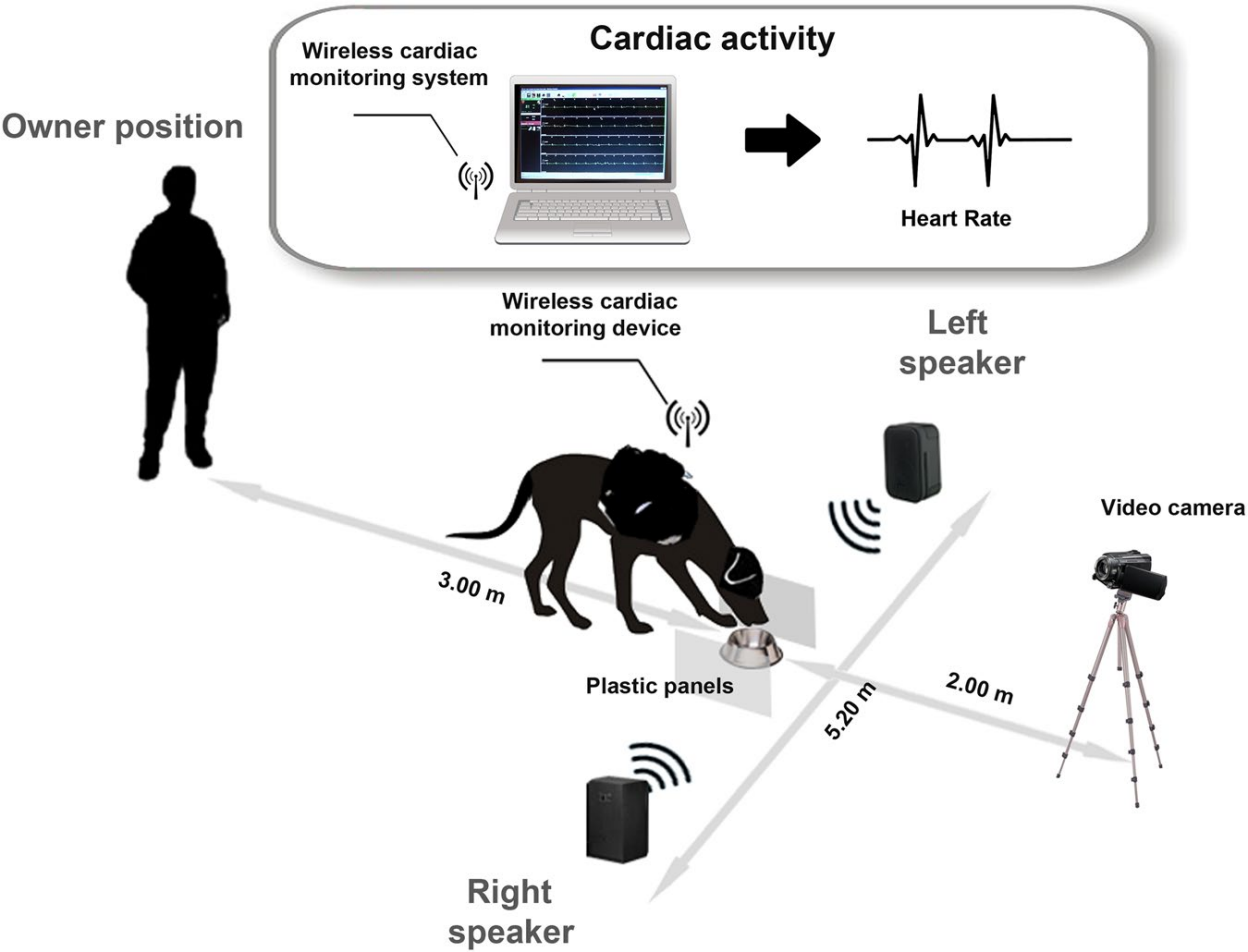
Experimental set-up. Schematic representation of the testing apparatus.

Experiment was carry out in an isolated room of the Department of Veterinary Medicine, University of Bari. Two speakers (FBT-200W8RA®) connected to a sound mixer were used to play simultaneously acoustic samples. A bowl, fastened to the floor with adhesive tape and full of dogs’ favorite food, was placed between the speakers, centrally (2,60 m from each speaker) and aligned with them. Furthermore, two plastic panels (30 cm high, 50 cm in depth) were located on the two side of the bowl at a distance of 30 cm, to help dogs to maintain a central position during the test (see Fig. 7).

A digital video camera was used to record dogs’ responses to acoustic stimuli. It was positioned on a tripod directly in front of the bowl, facing the subject and at a distance of about 2 m.

### Procedure

Each dog was presented with one of the three sets made up of the six basic emotional vocalizations (12 subjects per set). The playbacks’ order of each set was randomized between subjects. The test consisted of three weekly trials. In each trial two different vocalizations (one per emotion) were played.

The owner led the dog to the bowl on a loose leash. Once the subject took the right position (facing the video camera and centrally positioned between the two speakers) and soon after it started feeding, the owner let the dog off the leash and positioned himself 3 m behind the dog. Owners were instructed to stand still and not to interact with their dogs during the test. After 10 seconds from the owner positioning, the first stimulus was played. The two different vocalizations were played with at least 30 seconds interval between them. If after hearing the vocalization the subject did not resume feeding within this interval, the other playback was postponed. The maximum time allowed to resume feeding was 5 minutes. In the event of not resuming to feed before the session end, the missing vocalization was presented in the subsequent session.

Two experimenters from an adjacent room via a closed-circuit video system controlled stimuli playbacks. It consisted of a webcam, used to monitor the subjects’ reaction and position, and two computers (one inside the test room and the other outside it), connected by a local area network, to control the stimuli playbacks.

### Head-orienting response

First, a % Response index (%Res) for each dog head-orienting response to human vocalizations was calculated using the formula %Res = (L + R + NT/L + R + NT + N), where L and R signify respectively the number of Left and Right head-orienting responses, NT the number of times in which the dog stopped feeding without turning his head toward the speakers and N signifies “No response” (i.e. if the dog did not turn its head within five seconds after the playback). Given that dogs respond turning their head in different directions according to the emotional valence of the sound heard^17^, three responses were considered: turn right, turn left and no response, when the dog did not turn its head within 5 seconds from the sound playback. After a pilot test we decided to abandon the multiple presentation of the same acoustic stimulus since habituation to human vocalizations occurred very quickly. Lateral asymmetries in the direction of head-turning responses for each dog were scored as follows: a score of 1.0 represents head turning to the left side, −1.0 head turning to the right side and a score of 0 indicates no turns of the head.

### Behavior score

Dogs’ behavior was video recorded continuously throughout the experiment. Scores for stress/anxiety and affiliative behaviors were computed allocating a score of 1 for each behaviors displayed. A total of 28 behaviors were considered (see Suppl. Table 4 supplementary for the entire behavior list). The reactivity time (i.e. time elapsing from playback start and feeding stop) and the latency time (i.e. the time to resume feeding from the bowl after playbacks) were also measured; the maximum time allowed to resume feeding was 5 minutes.

For both, head-orienting responses and behavior scores, video footages were analyzed by two trained observers who were blind to the testing paradigm. The inter observer reliability was assessed by means of independent parallel coding of videotaped sessions and calculated as percentage agreement; percentage agreement was always more than 94%.

### Cardiac activity

The evaluation of dogs’ heart rate response during session was carried out following the methodology previously described by Siniscalchi and colleagues^32,35^. Briefly, the cardiac activity was recorded continuously during sessions, using the PC-Vetgard^+tm^ Multiparameter wireless system for telemetric measurements (see Fig. 7). The heart rate response was calculated from the onset of the sound and during the following 25 s. If the dog did not resume feeding within this interval, the heart rate response was analysed till it resumed to feed (maximum time allowed was 5 minutes). Dogs became accustomed to vests, keeping the electrodes in contact with their chest, during weekly visit to the laboratory before the experimental test until they showed no behavior signs of stress.

The heart rate (HR) curve obtained during the pre-experimental phase (ECG R-R intervals during the recording period) was used in order to calculate the HR basal average (baseline). The highest (HV) and lowest values (LV) of the HR response to different playbacks were scored. In addition, the area delimited by the HR curve and the baseline was computed for each dog and each sound separately using Microsoft Excel®. The Area Under Curve (above baseline and under curve, AUC) was then graphically separated from the Area Above Curve (under baseline and above curve, AAC). Each area value was then calculated and expressed as number of pixels (Adobe Photoshop Elite®). HR changes for each dog during presentations of different emotional vocalizations were then analyzed by comparing different area values with the corresponding baseline.

### Questionnaire

A modified version of the questionnaire, deriving from the Hsu and Serpell study^56^, was submitted to owners before the beginning of the session, in order to gather information on the canine-human relationship of their dogs (see Suppl. Table 5). Owners were asked to rate dogs’ response in a given situation on a four-point scale, where a score of zero represented no reaction to the stimulus while a score of four represented a strong reaction to it. The total score for each query was calculated by adding up the score obtained for each of the given situations.

## Statistical Analysis

### Head orienting response

Given that data for %Res were not normally distributed, the analysis was conducted by means of non-parametric tests (Friedman’s ANOVA).

A binomial GLMM analysis was performed to assess the influence of “emotion category”, “vocalization gender”, “playback order”, “sex” and “age” on the test variable: “head orienting response” with the “query scales” as covariants and “subjects” as random variable. To detect differences between the emotion categories Fisher’s Least Significant Difference (LSD) pairwise comparisons were performed. In addition, asymmetries at group-level (i.e. emotion category) were assessed via One-Sample Wilcoxon Signed Ranks Test, to report significant deviations from zero.

#### Reactivity and latency to resume feeding

For both reactivity and latency data, as they contained censored measurements, survival analysis methods were used^57^. Specifically mixed effects Cox regression modeling and Kaplan Meier estimates were used to analyze reactivity and the latency to resume feeding with the “emotion category” as the main factor (after a visual inspection of the data we decided to indicate “anger” as a reference category) and “subjects” as random variable. Mixed effects Cox proportional hazard models were used to analyze the effect of “vocalization gender”, “playback order”, “sex”, “age”, “Stress-behaviors” and “query scales” on the test variables: “reactivity” and “latency to resume feeding”.

### Cardiac activity and behavior score

GLMM analyses was performed to assess the influence of “emotion category”, “vocalization gender”, “playback order”, “sex” and “age” on the test variables: “HV”, “LV”, “AUC”, “AAC” and “Stress-behaviors” with the “query scales” as covariants and “subjects” as random variable. To detect differences between the emotion categories Fisher’s Least Significant Difference (LSD) pairwise comparisons were performed.

### Ethics statement

The experiments were conducted according to the protocols approved by the Italian Minister for Scientific Research in accordance with EC regulations and were approved by the Department of Veterinary Medicine (University of Bari) Ethics Committee EC (Approval Number: 3/16); in addition, before the experiment began, the procedure was explained to owners and written informed consent was obtained.

### Data availability

The datasets generated during and/or analysed during the current study are available from the corresponding author on reasonable request.

## Electronic supplementary material

Supplementary Information
