# Supplementary material for: Lateralized behavior and cardiac activity of dogs in response to human emotional vocalizations
Source: Sci Rep. 2018 Jan 8;8:77. doi: 10.1038/s41598-017-18417-4 (PMC5758824; doi:10.1038/s41598-017-18417-4)
Supplement: Supplementary file 1 — Supplementary Information [file 41598_2017_18417_MOESM1_ESM.doc]

**Supplementary Information**

**Title of manuscript:**

Lateralized behavior and cardiac activity of dogs in response to human emotional vocalizations..

**Authors:**

Marcello Siniscalchi^*1^, Serenella d’Ingeo^1^, Serena Fornelli^1^ and Angelo Quaranta^1^

^1^ Department of Veterinary Medicine, Section of Behavioral Sciences and Animal Bioethics, University of Bari “Aldo Moro”, Italy

^*^ Corresponding author

Correspondence to marcello.siniscalchi@uniba.it

**Supplementary Table 1. Subjects characteristics.**

| **Dog** | **Breed** | **Sex** | **Age** | **Neutered** |
| --- | --- | --- | --- | --- |
| **Argo** | Pointer | M | 10 | no |
| **Cudron** | Mongrel | M | 2 | yes |
| **Demon** | Mongrel | M | 4 | no |
| **Glengran** | Australian shepherd | M | 4 | no |
| **Morgan** | Bull terrier | M | 1 | no |
| **Popo** | Mongrel | M | 1 | no |
| **Raton** | Australian shepherd | M | 4 | no |
| **Rico** | French bulldog | M | 2 | no |
| **Russel** | Mongrel | M | 2 | no |
| **Schizzo** | Mongrel | M | 13 | yes |
| **Totò** | Mongrel | M | 8 | yes |
| **Woodstock** | Mongrel | M | 6 | no |
| **Zen** | Mongrel | M | 8 | no |
| **Bud** | Australian shepherd | M | 1 | no |
| **America** | Australian shepherd | F | 2 | no |
| **Bea** | Jack russel | F | 2 | no |
| **Bette** | Mongrel | F | 1 | no |
| **Chanel** | Dachsund | F | 3 | no |
| **Cheri** | Australian shepherd | F | 4 | yes |
| **Dea** | Irish setter | F | 5 | yes |
| **Kima** | Mongrel | F | 6 | yes |
| **Kira** | German shepherd | F | 2 | no |
| **Maia** | Mongrel | F | 3 | no |
| **Nika** | Mongrel | F | 4 | yes |
| **Sofia** | Mongrel | F | 2 | yes |
| **Stella** | Mongrel | F | 4 | no |
| **Tiffany** | Cocker spaniel | F | 4 | no |
| **Zana** | Mongrel | F | 4 | yes |
| **Zoe** | Mongrel | F | 2 | no |

**Supplementary Table 2. Questionnaire used to evaluate and classify playbacks of the six human basic emotions.**

**•** The emotion expressed is:

☐ Positive ☐ Negative

• Which emotion it represented?

☐ Disgust ☐ Happiness

☐ Fear ☐ Anger

☐ Surprise ☐ Sadness

• On a 3-point-scale, how clearly you perceived the emotion conveyed (1=minimum; 3=maximum)

☐ 1 ☐ 2 ☐ 3

The criterion for selecting the samples was as follows: a 90% agreement was used to select the vocalizations in the first instance using a match between the valence scoring and the type of emotion expressed (e.g. for joy= Positive + Joy; Fear= Negative + Fear). Then higher scores for the clarity of emotion conveying were used to select the final sample. If there were vocalizations with the same score, a random selection proceeded. For surprise vocalization, the selection criteria were the same except for the emotional valence score (i.e. positive-negative), which was not considered. Using a 90% agreement criterion, participants identified respectively 77 (out of 84) for “joy” vocalizations, 74 (out of 84) for “anger”, 70 (out of 84) for “fear”, 64 (out of 84) for “disgust”, 68 (out of 84) for “sadness” and 61 (out of 84) for “surprise”.

**Supplementary Table 3. Questionnaire used to evaluate and classify playbacks of the six human basic emotions.**

| Emotion | Set 1 | Set 2 | Set 3 |
| --- | --- | --- | --- |
| Fear | Male | Female | Female |
| Sadness | Female | Female | Male |
| Anger | Male | Female | Female |
| Happiness | Female | Male | Female |
| Surprise | Female | Male | Male |
| Disgust | Female | Male | Female |

**Supplementary Table 4. List of behaviors scored according to the Stress/Anxiety category.**

| **Behavioral Category** | **Scored Behavior** |  |
| --- | --- | --- |
| Stressed/Anxiety | ears held in tension  slightly spatulate tongue  tongue way out  braced legs  tail down-tucked  panting  salivating  look away of avoidance  flattended ears  head lowered  paw lifted  lowering of the body posture  vocalization  whining  shaking of the body,  running away  hiding  freezing  lips licking  yawning  splitting  blinking  seeking attention from the owner sniffing on the ground  turn away  height seeking posture |  |
| Affiliative | Tail wagging  Approach to the speaker |  |

**Supplementary Table 5. Questionnaire presented to owners in order to gather information about their dogs’ temperament and the dog-human relationship.**

| Item |
| --- |

*1-Stranger-directed aggression*

Dog acts aggressively

When approached directly by and unfamiliar male adult while being walked or exercised on a leash

When approached directly by and unfamiliar female adult while being walked or exercised on a leash

When approached directly by and unfamiliar child while being walked or exercised on a leash

Toward unfamiliar persons approaching the dog while it is the owner’s car

When an unfamiliar persons approaching the owner or a member of the owner’s family at home

When an unfamiliar persons approaching the owner or a member of the owner’s family away from home

When mailmen or other delivery workers approach the home

When strangers walk past the home while the dog is in the yard

When joggers, cyclists, roller skateboarders pass the home while the dog is in the yard

Toward unfamiliar persons visiting the home

*2-Owner-directed aggression*

Dog acts aggressively

When verbally corrected or punished by a member of the household

When toys, bones, or other objects are taken away by a member of the household

When bathed or groomed by a member of the household

When approached directly by a member of the household while it is eating

When food is taken away by a member of the household

When stared at directly by a member of the household

When a member of the household retrieves food or objects stolen by the dog

*3-Stranger-directed fear*

Dog acts anxious or fearful

When approached directly by an unfamiliar male adult while away from the home

When approached directly by an unfamiliar female adult while away from the home

When approached directly by an unfamiliar child adult while away from the home

When unfamiliar persons visit the home

*4-Non social fear*

Dog acts anxious or fearful

In response to sudden or loud noises

In heavy traffic

In response to strange or unfamiliar objects on or near the sidewalk

During thunderstorms

When first exposed to unfamiliar situations

In response to wind or wind-blown objects

*5-Separation-related behaviour*

Dog displays

Shaking, shivering, or trembling when left or about be left on its own

Excessive salivation when left or about to be left on its own

Restlessness, agitation, or pacing when left or about to be left on its own

Whining when left or about to be left on its own

Barking when left or about to be left on its own

Howling when left or about to be left on its own

Chewing or scratching at doors, floor, windows, and curtains when left or about to be left on its own

Loss of appetite when left or about to be left on its own

*6-Attachment or attention-seeking behaviour*

Dog

Displays a strong attachment for a particular member of the household

Tends to follow a member of household from room to room about the house

Tends to sit close to or in contact with a member of the household when that individual is sitting down

Tends to nudge, nuzzle, or paw a member of the household for attention when that individual is sitting down

Becomes agitated when a member of the household shows affection for another persons

Becomes agitated when a member of the household shows affection for another dog or animal

*7-Trainability*

Dog

Returns immediately when called while off leash

Obeys a sit command immediately

Obeys a stay command immediately

Will fetch or attempt to fetch sticks, balls, and other objects

Seem to attend to or listen closely to everything the owner says or does

Is slow to respond to correction or punishment

Is slow to learn new tricks or tasks

Is easily distracted by interesting sights, sounds, or smells

*8-Excitability*

Dog overreacts or is excitable

When a member of the household returns home after a brief absence

When playing with a member of the household

When the doorbell rings

Just before being taken for a walk

Just before being taken on a car trip

When visitors arrive at its home

*9-Pain sensitivity*

Dog acts anxious or fearful

When examined or treated by a veterinarian

When having its claws clipped by a household member

When groomed or bathed by a household member

_________________________________________________________________________________
